# Health and immunisation services for the urban poor in selected countries of Asia

**DOI:** 10.1186/s40249-019-0538-4

**Published:** 2019-04-18

**Authors:** John Grundy, Xiaojun Wang, Kunihiko Chris Hirabayashi, Richard Duncan, Dexter Bersonda, Abu Obeida Eltayeb, Godwin Mindra, Robin Nandy

**Affiliations:** 1 College of Public Health, Medicine and Veterinary Sciences, James Cook University, Cairns, Australia; 2 UNICEF East Asia and Pacific Regional Office, Bangkok, Thailand; 3 UNICEF Headquarter, New York, USA; 4 UNICEF, Manila, Philippines

**Keywords:** Urban health, Urban immunisation, Health equity, Governance, Disease control, Community engagement

## Abstract

**Background:**

Asia is a region that is rapidly urbanising. While overall urban health is above rural health standards, there are also pockets of deep health and social disadvantage within urban slum and peri-urban areas that represent increased public health risk. With a focus on vaccine preventable disease and immunisation coverage, this commentary describes and analyses strengths and weaknesses of existing urban health and immunisation strategy, with a view to recommending strategic directions for improving access to immunisation and related maternal and child health services in urban areas across the region. The themes discussed in this commentary are based on the findings of country case studies published by the United Nations Childrens Fund (UNICEF)  on the topic of immunisation and related health services for the urban poor in Cambodia, Indonesia, Mongolia, Myanmar, the Philippines, and Vietnam.

**Main body:**

Although overall urban coverage is higher than rural coverage in selected countries of Asia, there are also wide disparities in coverage between socio economic groups within urban areas. Consistent with these coverage gaps, there is emerging evidence of outbreaks of vaccine preventable diseases in urban areas. In response to this elevated public health risk, there have been some promising innovations in operational strategy in urban settings, although most of these initiatives are project related and externally funded. Critical issues for attention for urban health services access include reaching consensus on accountability for management and resourcing of the strategy, and inclusion of an urban poor approach within the planning and budgeting procedures of Ministries of Health and local governments. Advancement of local partnership and community engagement strategies to inform operational approaches for socially marginalised populations are also urgently required. Such developments will be reliant on development of municipal models of primary health care that have clear delegations of authority, adequate resources and institutional capabilities to implement.

**Conclusions:**

The development of urban health systems and immunisation strategy is required regionally and nationally, to respond to rapid demographic change, social transition, and increased epidemiological risk.

**Electronic supplementary material:**

The online version of this article (10.1186/s40249-019-0538-4) contains supplementary material, which is available to authorized users.

## Multilingual abstracts

Please see Additional file 1 for translations of the abstract into the five official working languages of the United Nations.

## Background

Rapid urbanisation is a dominant demographic and social trend in Asia. As of 2010, 45.6% of the population in the Asia Pacific is living in urban areas, and this figure is projected to increase to 50% by 2026 [1]. This is presenting a major challenge for government and civil society partners, who are struggling to adapt public service and social protection systems to the social conditions arising from demographic transition, increased urbanisation and increasing population mobility. Modern health sectors, which have been traditionally based on a rural model of primary health care, are endeavouring to adapt governance, financing, and service delivery arrangements to the needs of urban populations, particularly of the urban migrants and poor. The overall number of slum dwellers in low and middle-income countries is expected to double from one billion to two billion globally by 2030 [2]. Based on published population data from 2016, there is an estimated 63 million people who are living in slum communities in the six countries being discussed in this commentary [3]. These countries include Cambodia, Indonesia, Mongolia, Myanmar, the Philippines, and Vietnam.

One of the health programmes that needs to adapt to this rapid transition are national immunisation programs, which have been highly successful in administering essentially rural based programs for prevention and control of vaccine preventable diseases such as neo natal tetanus, polio, and measles over the last 30 years [4]. But the recent emergence between 2014 and 2016 of substantial vaccine preventable disease outbreaks in major cities of the region including Ulaanbaatar, Yangon, Manila, and Hanoi, along with evidence emerging of substantial coverage gaps between wealth quintiles in urban areas, are raising questions now about the effectiveness and equity of immunisation programming in rapidly urbanising areas.

So just how are countries in the region adapting national programs to the realities of this rapidly urbanising context? In a recently published set of case studies and thematic analyses on this topic, the United Nations Children’s Fund (UNICEF) reviewed the policy and planning responses of countries to urban health and immunisation in selected countries of the Asian region [5]. Table 1 below summarises the timing, data sources and major findings from the case studies.

**Table 1:** Timing, data sources and main findings of case studies in health and immunisation in East Asia

Timing	The case studies were developed in 2017, and examined epidemiological, demographic and social trends over the last 15 years.
Data sources	The main sources of information for the case studies included published literature about health and immunisation in urban settings, relevant national health policy and planning documents, and data bases of the World Health Organisation, Demographic and Health surveys, Multi Indicator cluster surveys and UN-HABITAT population data.
Summary of main findings	○ There is rapid urban growth in all reviewed countries, not only in major cities, but also in provincial and district capitals, with significant numbers residing in urban slums. ○ In five out of the six countries, coverage gaps between the highest and lowest wealth quintiles for urban populations are quite significant. ○ All six countries are reporting vaccine preventable disease outbreaks in urban areas in recent years. ○ Social conditions expose the urban poor to higher risk of vaccine preventable disease outbreak. ○ There have been some innovations in operational responses to lower coverage amongst the urban poor, but implementation sustainability is constrained by externally financed projects operating in highly complex local governance contexts.

As well as detailing the main findings, this commentary will suggest potential policy and planning responses for improving coverage and equity of access, with a focus on sustainability of interventions.

## Main text

### Social conditions and demographic trends amongst the urban poor in Asia

In Myanmar, Vietnam, the Philippines, Cambodia, and Mongolia, access to government services is regulated through systems of civil registration. Although health systems are intended to provide services to all the population, lack of registration by urban migrants can restrict access through limited financing of health services for non-registered populations. It may also serve to restrict demand, as lack of legal status may inhibit the very poor from accessing public services due to concerns regarding the cost of services for unregistered populations [6]. In Mongolia and the Philippines for example, social sector administrators and local authorities were requested to link up with the immunisation strategy to expand population access to services [7]. In Vietnam, lack of registration of new arrivals in Ho Chi Minh City has meant that there has been a chronic underestimate of the ‘real’ population in the catchment area, with surveys subsequently confirming low overage amongst the urban poor [8]. The question as to whether rural to urban poor migrants are being counted and included in health and local government registers is therefore a major issue to be addressed in urban health policy.

Compounding this problem of the hidden nature of the urban poor is the severity of the social conditions in which they reside. There are large agglomerations of poor who relocate to areas where there is limited public health infrastructure and services, such as in the peri urban areas of Yangon and Phnom Penh. In Cambodia, the urban poor reside both in these peri urban areas, as well as in inner urban areas along railway tracks, in less accessible high-rise buildings and on public or privately-owned lands [9]. In Myanmar, there are very high concentrations of peri urban poor, who have flooded into the city post political reform seeking improved economic opportunity [10].

**Fig. 1:**
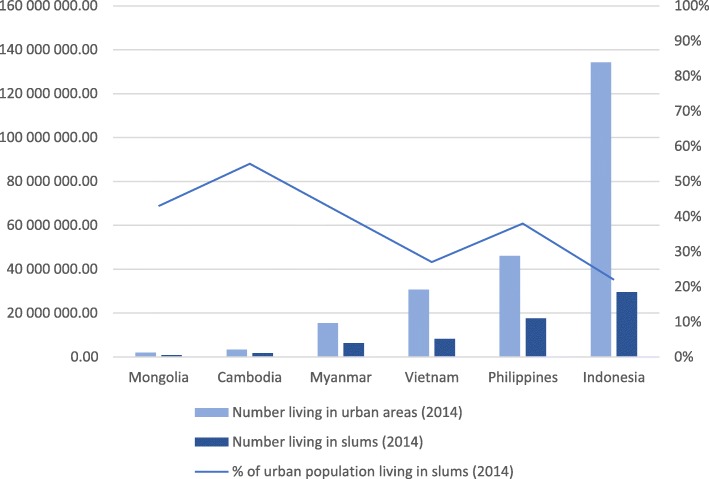
Numbers and percent living in urban areas and slums 2014. Data sourced from [42]

As outlined in Fig. 1, substantial proportions of these urban populations are living in urban slums.

The UN-HABITAT definition of slums includes assessment of five major characteristics that include access to improved drinking water sources and sanitation facilities, durability of housing, enough living areas, and security of tenure [11]. Some of these criteria are also applied to assess household wealth in Demographic and Health Surveys (DHS). In the 2014 Cambodia DHS Survey, criteria such as household dwelling and household characteristics, consumer goods, and assets are used as a measure of socioeconomic status, after which standardized scores are used to define wealth quintiles [12]. These classifications provide a degree of consistency in the measurement of the severity of social conditions in urban poor areas, especially given the diversity of social backgrounds that make up slum populations relating to socioeconomic status, occupation, ethnicity, and levels of mobility [13].

### Demographic trends in urban areas

The case studies demonstrate rapid growth in urbanisation across the region, with these demographic trends set to continue. In Mongolia, the political and free market reforms also unleashed rapid rural to urban migrations, resulting in the relocation of dispersed traditional housing settlements in rural regions to very high concentrations of these settlements in the ‘Ger Districts’ of Ulaanbaatar [14]. This rural style of living in the congested urban areas presents overwhelming public health infrastructure challenges relating to water, sanitation, and access to public services. Two out of the six countries will be more than 50% urbanised by 2025, and Indonesia by 2015 is already mostly urbanised. In five out of six countries, urbanisation trends are still on the increase [15].

**Fig. 2:**
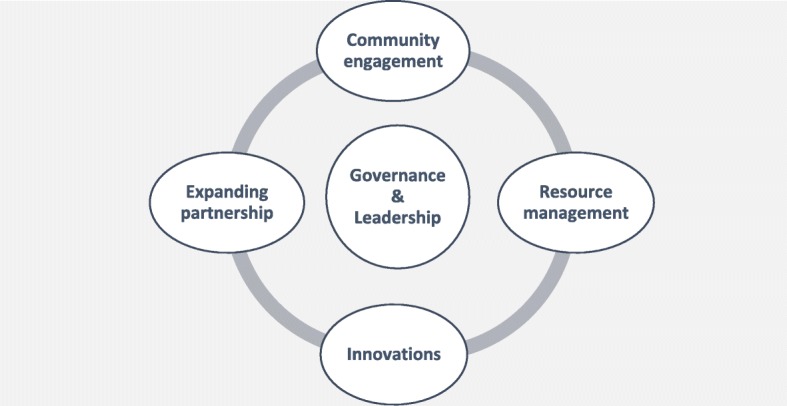
Building the enabling environment for improving urban health systems

This urban growth is occurring not only in the capitals of each country, but also in provincial cities and district towns, particularly in the larger population countries. Indonesia now has 27 cities and towns with populations of greater than 300 000. In the Philippines, this figure has now reached 18 cities and towns [15]. In the larger population countries, development of a nationwide urban development strategy is therefore an imperative.

Evaluation studies of immunisation program initiatives in Mongolia [16] and Cambodia [9], and reports from Vietnam [8] and Myanmar [17] indicate the problem of rapid migration to peri urban areas results in agglomerations of unregistered and uncounted populations, who either are not accessing services, or who are not being included within population denominators. These observations from the case studies align with those of a recent systematic review of urbanisation and immunization, which found that rural to urban migration has a negative effect on coverage in such settings as India, China and Nigeria, with processes of migration limiting the capability of parents to access health care services [18]. The case studies demonstrate that there are high levels of vulnerability in these migrant populations, due to lack of civil registration and insecure land tenure, which contributes to a sense of lack of entitlement to health care. Other dimensions of vulnerability include high levels of mobility, and lack of knowledge of vaccine preventable diseases and the benefits of vaccination [19].

### Immunisation coverage and vaccine preventable diseases in urban areas

The case studies identified that coverage gaps between wealth quintiles within urban areas are significant in five out of six cases, with a coverage gap in Cambodia of 12.1% between highest and lowest wealth quintiles in 2014, an 18.9% gap between highest and lowest wealth quintiles in Indonesia in 2012, a 16.7% gap between wealth quintiles in the Philippines in 2013, and an 11% gap between wealth quintiles in Vietnam in 2013. The highest gap between wealth quintiles in this group of countries is a gap of 29.2% in Myanmar in 2015.

Despite evidence of these gaps, analysis of data from successive DHS surveys (for children aged 12–59 months) indicates some narrowing of inequities. In Cambodia, analysis on urban coverage over the last 14 years has demonstrated a narrowing of inequities between wealth quintiles, where coverage gaps between the highest and lowest wealth quintiles (DPT3) have narrowed from 49% in 2000 to 12.2% in 2014 (children aged 12–59 months). The case study attributes this narrowing in coverage gaps to implementation of an EPI Review in 2010, which identified socially marginalised groups such as ethnic minorities and the urban poor as being at high risk for un-immunised status. These evaluation findings then translated into a nationwide “high risk strategy” that aims to identify high-risk groups and their locations across the country. A published review of the recent history of immunisation coverage in Cambodia identified nationwide application of the Reaching Every District strategy as a main factor in coverage improvement and recommended a more granular sub district micro-planning operational strategy to reach the last 10–20% of the unvaccinated population [20].

Coverage gaps in Indonesia between highest and lowest wealth quintiles for DPT3 have narrowed from a 26% gap in 1997 to a 19% gap in 2012. In the Philippines, the same gap has narrowed only slightly from 17.3% in 1993 to 15.5% in 2013 (DPT3).

### Evidence of vaccine preventable disease outbreaks in urban areas in Asia

The case studies illustrate that recent vaccine disease outbreaks in Asia provide additional evidence to support the claim of significant immunity gaps in urban populations. In 2014 in the Philippines, there was a measles outbreak with highest caseloads of measles in the National Capital Region [7]. A diphtheria outbreak then occurred in 2015 with 105 cases, of which one third of cases were reported in one City Hospital in Metro Manila [21]. In 2015 in Vietnam, there was a nationwide measles outbreak, with 150 deaths reported at the National Paediatric Hospital in Hanoi [8]. In Indonesia, there have been annual measles outbreaks in slums in North, East and West Jakarta Districts which comprised two thirds of annual confirmed measles cases [22]. In Phnom Penh in 2005, a vaccine derived polio virus was detected in an urban slum area near the centre of the city, which resulted in the need for a subsequent national campaign effort [13]. And finally, in Mongolia in 2016, there was a measles outbreak nationally, with the outbreak first reported in Ulaanbaatar City and most cases reported from there. In all there were 23 000 cases and 105 fatalities [23].

These findings point to significant immunity gaps for vaccine preventable diseases in urban settings in Asia and align with other findings of the risks of vaccine preventable disease outbreaks in urban areas. A study of measles incidence in urban slums of Surat City in Gujarat India observed that urban slums are at particular risk of rapid disease transmission, due to overcrowding and lower vaccination rates. Urban poor populations can provide a reservoir for the measles virus which can then be transmitted more widely to rural populations [24]. Along with public health factors such as lack of hygiene, malnutrition, overcrowding and high illiteracy in slums, health system factors such as low immunization coverage and poor surveillance are also responsible for disease outbreaks in India [25].

Inequities in urban immunisation coverage are not only related to measles and diphtheria in urban areas. The same coverage inequities are likely to be evident in relation to the introduction of newer vaccines such as pneumococcal and rotavirus vaccines. Recent data from the 2017 Demographic Health Survey in the Philippines demonstrates that the wealthiest urban health quintile has a coverage of 62% for the first dose of pneumococcal vaccine, whereas the lowest urban wealth quintile has coverage of 45% [26]. Given that studies in urban hospitals in Myanmar [27], Cambodia [28] and Mongolia [29] have demonstrated that rotavirus is the leading cause of severe acute gastroenteritis as well as diarrhoea related hospitalizations for children less than 5 years of age, equity of access to new vaccines for the urban poor (including for pneumococcal and human papillomavirus vaccines) should be a high policy and planning priority for urban health planners.

### Innovations in immunisation operational strategy in urban areas

In the cases of Vietnam, Cambodia, Mongolia, Myanmar, the Philippines and Indonesia, national programs have been trialling approaches to urban immunisation strategy. In Cambodia, building on lessons learned from campaign methodology and a reaching every community approach, the national program developed a ‘high risk community’ listing of villages across the country which included urban poor locations [30]. In Indonesia, a pilot project in Jakarta is trialling communication, monitoring and management strategies in high risk areas of Jakarta, with some promising coverage improvement reported [31]. Mongolia developed a revitalised Reaching Every District strategy from 2010, focusing on high risk identification, household visits to the urban poor, and social sector and civil society collaborations to expand access through improved civil registration. Several evaluations there have demonstrated the potential of the approach to improve immunisation and other health service access for the urban poor [6, 13]. In Myanmar, a reaching every community approach was designed in 2010, with a focus on mapping of high-risk areas, and an emphasis on analysis of risk in terms of social distance and not just physical distances [32].

In the Philippines, a “reaching every purok” strategy has been devised, as a corollary to a wider nationwide reaching every barangay approach (with purok and barangay referring to the lowest administrative levels in rural and urban locations respectively). Main elements of the strategy here include mapping of high-risk areas, identifying high risk puroks through household card checks, developing a method for risk classification, working with community volunteers and local authorities to expand access to services through household visits [33]. In Vietnam, a national immunisation review focussed on urban strategy development and highlighted major issues such as population tracking, waste management, public and private sector coordination, consistency in immunisation scheduling, and financing by local government as the main issues to be addressed in the upcoming comprehensive multi-year plan (cMYP) for immunisation [8].

Although project designs and financing have the potential to positively influence policies and plans, in the end, sustaining such project initiatives is reliant on their integration into national planning and budgeting systems. The Mongolia evaluations express in some detail the challenge in moving from a project to a system approach. Despite even the development of a Ministerial Decree on Reaching Every District strategy, the strategy has still failed to be embedded in health system planning and budgeting functions, leaving the initiative highly exposed to the vagaries of project financing [13]. Despite this managerial challenge, one in depth evaluation in Mongolia found that additional families can be reached through community level mapping and follow up, provided additional resources can be mobilised to reach out to the most disadvantaged [6].

Although some countries have documented separate guidelines for urban strategy (as in Cambodia, the Philippines, and Vietnam), the case studies identify that no country provides strategic or operational planning guidance for urban immunisation within a multi-year plan for immunisation. In Cambodia, the nationwide Reaching Every Community approach is being financed through the Global Vaccine Alliance (GAVI) Health System Strengthening funds, leaving the question open as to how national and local governments will take up financing of the strategy post project completion. In Vietnam, the clear evidence base and intent for strategy development is there, but the exact policy or procedural instrument by which to extract budgetary commitments from local Governments in Hanoi and Ho Chi Minh City remains unclear. [8] In the Philippines, advocacy meetings with local chief executives are proposed to accelerate the strategy, but how local governments are held accountable for financing and results are not made clear [16]. In Myanmar, the government has recently proposed significant decentralisation reforms in line with the new constitution. Despite these reforms, townships (the third administrative level in Myanmar) do not yet have operational budgets linked to health plans. In contrast, financing is ‘project based’ and strongly co related to external financing [34].

In summary, although there are promising innovations in operational strategy in urban immunisation, there are significant gaps in strategic policy and planning initiatives to tie down accountability and financing within policy or strategic planning documents, or in health system procedures.

### Governance arrangements for immunisation and urban health care in Asia

In the 1970s and 1980s, many of these countries including Myanmar, Mongolia, and Vietnam, built a primary health care system based on a rural health model, with clear command and control structures, and administrative boundaries with defined population catchments, linked to primary care centres and village volunteers networked into a ‘social mobilisation’ communication approach. The case studies demonstrate that urban health has evolved in a significantly more complex way, with governance and service delivery mechanisms dispersed across government, local government, civil society, and private sector actors, with a completely different set of institutional and population dynamics.

Local governments have also proved to have critical roles in urban immunisation. In Vietnam, whilst the central government retains its financing functions for vaccines and cold chain equipment, the financing of operations rests with local government officials [8]. In Myanmar, the Township Administrator (through the Township Health Committee) is responsible for ensuring the smooth operations of health services [35]. Since 2004, the Government of Cambodia legislated an ‘Organic Law’ which defined the decentralised roles of local government in the provision of health care. A published plan for the development of the Municipality of Phnom Penh envisions the expansion of public health services as coming within its area of governance accountability [36].

In the Philippines, services have been devolved to Local Government Units (LGUs) since the early 1990s, with these LGUs providing most of the operational financing of primary health care provision. In Mongolia, as part of its program of health reform, performance contracts were established between Family Group Practices, local government, and the Ministry of Health for provision of primary care [6]. In Indonesia, the central government has the responsibility for policy and guidelines, supplementary immunization activities, procurement of vaccines and syringes, and technical assistance. But it is local government that has the responsibility for implementation in their area, provides budget for human resource recruitment and management, including for incentives, transportation, and maintenance [37]. Added into this governance and service delivery mix are the activities of non-government organisations and the private medical sector, who both have key roles in primary health care in urban settings. Across the region, non-government organisations have been active in promoting access to primary care for the urban poor. Although the public sector remains the mainstay of immunisation service provision, there is also growing evidence of increased availability of vaccines through a private sector model as illustrated by the cases of Cambodia [38] and Vietnam [8].

Although these developments in devolution, and civil society and private sector development have also occurred in rural areas, it is in urban areas that civil reforms and private sector development has the highest concentration of reform initiatives and resources. The fragmentation of resource inputs, service providers and management arrangements will necessitate a coordinated partnership effort and cross sectoral collaboration to ensure efficiency and equity in essential public service delivery for urban poor areas. Similar findings were reached recently in a review of published studies on vector control in urban settings. This review concluded by recommending a joint approach involving policy makers and scientists, along with a call for increased political commitment and citizen engagement strategies in order to design an optimal response to vector control in urban settings [39].

### Potential policy and planning pathways for the organisation and management of health care services for the urban poor

Given the institutional complexity of urban health care outlined in the case studies, and considering the increased epidemiological risk associated with urban slum settlements, there is a strong case for a rethinking of the organisational and management frameworks for urban health care in Asia. Although there are indications that various service delivery trials and pilots across the six countries have developed promising technical and operational capabilities, issues surrounding the enabling environment for the organisation of urban health care are less clear. Figure 2 outlines various aspects of this enabling environment for urban health management, that would need to be addressed in order to develop sustainable management and delivery systems.

Defining the governance arrangements and accountabilities to support this enabling environment will be critical in terms of transitioning urban immunisation strategies from externally supported projects to core health system or local government functions. The urban immunisation strategy should define the governance arrangements, where by clear accountabilities from each set of stakeholders are defined in terms of overall coordination, human resource commitments, implementation of service delivery strategies and immunisation schedules, reporting of disease outbreaks, financing of operational budgets, maintenance of cold chain systems, waste management and communication and risk management. Community engagement strategy is also a critical component of governance, given the weak political position occupied by urban slum dwellers, and particularly those occupying land with insecure tenure.

Linking of urban immunisation strategy to health and social policy will be another critical step in supporting an enabling environment for urban health system development*.* As demonstrated by the cases of Vietnam, Mongolia, Myanmar and Cambodia, lack of registration with local authorities means that either populations have the perception they do not have entitlements to health care, or services are not funded to provide care to non-registered populations, or migrant populations are not included within population denominators. Linkage to social sector policies, protection mechanisms and registration systems therefore provides important opportunities for immunisation programs to leverage institutional supports from parallel social sector investments and strategies. Examples of these include health financing and social protection schemes for the poor, conditional cash transfers, and civil, birth and school registration systems. Better linking of immunisation micro-planning upwards to City Master Planning should also enable immunisation planners to integrate their programs into local government planning processes, and in doing so, become less reliant on what is emerging as a regional pattern of external time bound project financing and pilot projects.

These directions align with the recent findings of an examination of capacity for decentralised health management in the Philippines. This study observed that capacity for decentralized management by local authorities is reliant on a synergistic relationship between decision making space, capability, and accountability [40]. That, it is difficult to be accountable for performance in the absence of decision-making authority and adequate human or material resources to implement on a wider scale. Conversely, if the motivation for accountability is not in place, decision making authority may not be exercised and resources not allocated. Along with national level laws, policies and resources, local government leadership and governance should therefore also be at the centre of efforts to develop sustainable and effective urban health systems.

These conclusions also align with the main findings of a study of intra urban inequities in Bangladesh, which found that intra-urban inequities have narrowed for access to family planning and maternal care, largely due to improved access of slum populations to a variety of public sector, non-government and private sector primary health care clinics. Given this complexity of service provision, researchers concluded that it is vital to increase the government’s regulatory capacity and stewardship role in urban health, in order to ensure adequacy of resources, and quality and affordability of urban health care [41].

## Conclusions

The case studies on health and immunisation access in selected countries of Asia found that there are pockets of deep disadvantage in urban areas, with low immunisation coverage being associated with lower socio-economic status. These pockets of low coverage are resulting in widespread urban preventable disease outbreaks in Asia, and do not augur well for future containment of emerging diseases. It will be critical that policy makers tie down accountability for resourcing of the strategy, and to include the approach within the planning and budgeting procedures of Ministries of Health and local governments. In the medium to long term, technical agencies and partner governments will need to focus on development of municipal models of primary health care that have clear delegations of authority, adequate resources and institutional capabilities to implement. The development of urban health and immunisation strategy is urgently required regionally and nationally, to respond to the impacts of rapid demographic change, social transition, and increased epidemiological risk.

## Additional file

Additional file 1: Multilingual abstracts in the five official working languages of the United Nations. (PDF 776 kb)

## Abbreviations

CMYPs: Comprehensive multi Year Plan for Immunisation
DHS: Demographic and Health Survey
DPT: Diphtheria, Pertussis, and Tetanus Vaccine
GAVI: Global Vaccine Alliance
LGU: Local government unit
MICS: Multi Indicator Cluster Survey
UN: United Nations
UNICEF: United Nations Children’s Fund
WHO: World Health Organisation
