# Supplementary material for: Health and immunisation services for the urban poor in selected countries of Asia
Source: Infect Dis Poverty. 2019 Apr 18;8:26. doi: 10.1186/s40249-019-0538-4 (PMC6471782; doi:10.1186/s40249-019-0538-4)
Supplement: Supplementary file 1 — Multilingual abstracts in the five official working languages of the United Nations. (PDF 776 kb) [file 40249_2019_538_MOESM1_ESM.pdf]

Translation of the abstract into the five official working languages of the United Nations

خدمات الصحة والتحصين لفقراء الحضر في بلدان مختارة من آسيا

John Grundy, Xiaojun Wang, Kunihiko Chris Hirabayashi, Richard Duncan, Dexter Bersonda, Abu Obeida Eltayeb, Godwin Mindra, Robin Nandy

نبذة

الخلفية: آسيا هي منطقة تتوسع بسرعة. في حين أن الصحة الحضرية العامة أعلى من المعايير الصحية الريفية ،هناك أيضا جيوب من العيوب الصحية والاجتماعية العميقة داخل المناطق الحضرية الفقيرة والمناطق المحيطة بالمدن ، والتي تمثل مخاطر متزايدة على الصحة العامة. مع التركيز على الأمراض التي يمكن الوقاية منها باللقاحات والتحصين ، يصف هذا المحتوى ويحلل نقاط القوة والضعف في استراتيجية الصحة والتحصين الحضرية القائمة ، بهدف التوصية باتجاهات استراتيجية لتحسين الوصول إلى خدمات التحصين وما يتصل بها من خدمات صحة الأم والطفل في المناطق الحضرية في جميع أنحاء المنطقة. تستند الموضوعات التي نوقشت في هذا المحتوى على نتائج دراسات حالة البلد، نشره صندوق الأمم المتحدة الدولي لحالات الطوارئ للأطفال بشأن موضوع التحصين والخدمات الصحية ذات الصلة لصالح فقراء الحضر في كمبوديا وإندونيسيا ومنغوليا وميانمار والفلبين وفيتنام.

العرض: على الرغم من أن التغطية الحضرية الشاملة أعلى من التغطية الريفية في بلدان مختارة من آسيا ، فهناك أيضا تباينات واسعة في التغطية بين المجموعات الاقتصادية الاجتماعية داخل المناطق الحضرية. وتمشيًا مع هذه الفجوات في التغطية، هناك أدلة جديدة على تفشي أمراض يمكن الوقاية منها باللقاحات في المناطق الحضرية. استجابة لهذه المخاطر الصحية العامة المرتفعة، هناك بعض الابتكارات الواعدة في الاستراتيجية التشغيلية في المناطق الحضرية، على الرغم من أن معظم هذه المبادرات ذات صلة بالمشروعات وممولة من الخارج. وتشمل القضايا الحرجة التي يجب الاهتمام بها للوصول إلى الخدمات الصحية الحضرية التوصل إلى توافق في الآراء بشأن المساءلة عن الإدارة وتوفير الموارد للاستراتيجية، وإدراج نهج فقير في المناطق الحضرية ضمن إجراءات التخطيط والميزنة في وزارات الصحة والحكومات المحلية. كما أن هناك حاجة ماسة إلى النهوض بالشراكات المحلية وإستراتيجيات المشاركة المجتمعية لإرشاد النُهج التشغيلية للسكان المهمشين اجتماعياً. وستعتمد هذه التطورات على تطوير نماذج البلدية للرعاية الصحية الأولية التي لديها تفويض واضح للسلطة والموارد الكافية والقدرات المؤسسية اللازمة لتنفيذها.

الاستنتاج: إن تطوير النظم الصحية الحضرية واستراتيجية التحصين مطلوب على المستوى الإقليمي والوطني ، من أجل الاستجابة للتغير الديموغرافي السريع، والانتقال الاجتماعي، وزيادة المخاطر الوبائية.

Translated from English version into Arabic by Aalya Al-Beeshi, proofread by Asmaa Elkordy, through

TRANSLATORS
WITHOUT BORDERS

亚洲部分国家城市贫困人口享有的卫生和免疫服务

John Grundy, Xiaojun Wang, Kunihiko Chris Hirabayashi, Richard Duncan, Dexter Bersonda, Abu Obeida Eltayeb, Godwin Mindra, Robin Nandy

摘要

引言：亚洲是一个快速城市化的地区，城市总体卫生水平高于农村，但城市贫民窟和城郊地区尚存在严重的卫生和社会劣势，是城市公共卫生的隐患。本文聚焦于疫苗可预防疾病及其免疫接种的覆盖率，分析了城市当前的卫生和免疫接种策略的优缺点，以期为城市地区免疫接种覆盖率的提高，及相关妇幼卫生服务的改善提供策略建议。本评论对联合国儿童基金会涉及的柬埔寨、印

度尼西亚、蒙古、缅甸、菲律宾和越南等城市贫困人口的免疫接种和相关卫生服务发表的国家案例进行讨论。
主要内容：虽然亚洲部分国家的城市公共卫生覆盖率高于农村，但城市不同社会经济群体间的覆盖率存在较大差异。最新研究显示，城市地区暴发了疫苗可预防疾病，疫情与公共卫生覆盖差距呈正相关。为了应对这一增加的公共卫生风险，城市环境的运营策略现已作了一些创新，尽管这些举措大多与项目相关，且由外部提供资金。城市卫生服务需要注意的关键问题包括就策略的管理和资源问责制达成共识，将城市贫困问题纳入卫生部和地方政府的规划和预算程序。同时需要促进地方的伙伴关系和社区的策略参与度，为社会边缘化人口的工作提供信息。以上各方面的发展将依赖于具有明确授权、充足资源和机构实施能力的市政初级保健模式的发展程度。
结论：为应对人口、社会转型的快速变化以及流行病风险的增加，需要在区域和国家层面分别发展城市卫生系统和制定免疫策略。

Translated from English version into Chinese by Fan Yang, edited by Pin Yang

CHINESE CENTER FOR DISEASE CONTROL AND PREVENTION
NATIONAL INSTITUTE OF PARASITIC DISEASES

Services de santé et de vaccination pour les populations urbaines démunies dans certains pays d’Asie

John Grundy, Xiaojun Wang, Kunihiko Chris Hirabayashi, Richard Duncan, Dexter Bersonda, Abu Obeida Eltayeb, Godwin Mindra, Robin Nandy

RÉSUMÉ

Contexte: L’Asie est une région qui connaît une urbanisation rapide. Bien que la situation sanitaire des populations urbaines soit meilleure que celle des zones rurales, il reste des poches très défavorisées, du point de vue sanitaire et social, dans les bidonvilles urbains et péri-urbains, qui représentent un risque accru pour la santé publique. Axé principalement sur les maladies évitables par la vaccination et la couverture vaccinale, le présent commentaire décrit et analyse les points forts et les points faibles des stratégies existantes de santé et de vaccination en zones urbaines, dans le but de préconiser des orientations stratégiques permettant d’améliorer l’accès à la vaccination et aux services connexes de santé de la mère et de l’enfant dans les zones urbaines de la région. Les thèmes abordés ici se fondent sur les résultats d’études de cas de pays publiées par le Fonds international d’urgence des Nations Unies pour l’enfance (UNICEF) sur le sujet de la vaccination et des services de santé connexes à destination des populations urbaines pauvres au Cambodge, en Indonésie, en Mongolie, au Myanmar, aux Philippines et au Vietnam.
Discussion: Bien que la couverture sanitaire soit meilleure dans les villes que dans les zones rurales des pays asiatiques choisis, il existe de larges disparités entre les groupes socioéconomiques dans les zones urbaines. Ces lacunes de la couverture coïncident avec l’apparition de foyers épidémiques de maladies évitables par la vaccination dans les zones urbaines. En réponse à ces risques de santé publique accrus, des stratégies opérationnelles novatrices et prometteuses ont été déployées dans les zones urbaines, mais la plupart du temps en lien avec des projets et bénéficiant de financements externes. Les questions cruciales concernant l’accès aux services de santé en milieu urbain comprennent, d’une part, l’établissement d’un consensus sur la responsabilité de la gestion et des ressources de la stratégie et, d’autre part, l’inclusion d’une approche des populations urbaines démunies dans les procédures de

planification et budgétaires des ministères de la santé et des autorités locales. Il est également nécessaire et urgent de déployer des stratégies de partenariats locaux et d'implication des populations, afin d'informer des approches opérationnelles visant les populations en marge de la société. Ces évolutions dépendront de l'élaboration de modèles municipaux de soins de santé primaires prévoyant une délégation claire des pouvoirs, des ressources adéquates et les capacités institutionnelles nécessaires à leur réalisation.

Conclusions: L'élaboration de systèmes de santé urbains et de stratégies vaccinales est nécessaire au niveau régional et national afin de répondre aux changements démographiques rapides, à la transition sociale et à l'augmentation des risques épidémiologiques.

Translated from English version into French by Suzanne Assenat, proofread by Gwenaelle Le Jan-Moulart, through

TRANSLATORS WITHOUT BORDERS

Услуги здравоохранения и вакцинации для городской бедноты в отдельных странах Азии

Джон Гранди, Ксяо Джан Уэнг, Кунишико Крис Хирабайаши, Ричард Дункан, Декстер Берсонда, Абу Обейда Эльтайб, Гудвин Майндра, Робин Нанди.

АННОТАЦИЯ

Введение: Азия –это быстро урбанизирующийся регион. Несмотря на то, что общие стандарты городского здравоохранения превышают сельские, существует большое количество проблем, касающихся здравоохранения и социального развития в городских трущобах и пригородных районах, которые представляют большой риск для здоровья общественности. Обращая внимание на вакциноуправляемые инфекции и распространенность вакцинации, в данной записке представлен анализ плюсов и минусов существующей стратегии городского здравоохранения и вакцинации с целью предложить стратегические указания для улучшения доступа к вакцинации и сопутствующим службам здоровья матери и ребенка в городских районах по всему региону. В основе данного отчета лежат результаты предметных исследований по стране, опубликованных Детским фондом ООН по вакцинации и сопутствующим услугам здравоохранения для городской бедноты Камбоджи, Индонезии, Монголии, Мьянмы, Филиппин и Вьетнама.

Основная часть: Несмотря на то, что, в целом, в указанных странах Азии городские масштабы превышают сельские, наблюдается существенный дисбаланс в положении социально-экономических групп внутри городских районов. В соответствии с этими различиями, возникают случаи заболеваемости вакциноуправляемыми инфекциями в городских районах. В ответ на растущий риск заболеваемости, были проведены изменения действующей стратегии в городских поселениях, хотя многие из существующих инициатив относятся к проекту и финансируются извне. К ключевым решениям по привлечению внимания к доступности городских служб здравоохранения относятся: достижение компромисса по вопросам ответственности за финансовое обеспечение и управление стратегией, а также включение подхода к городской бедноте в рамках планирования и бюджетирования Министерством здравоохранения и

муниципальными властями. Также имеется острая необходимость в улучшении стратегий местного партнерства и группового вовлечения с целью извещения об операционных подходах для социально-маргинальных слоев населения. Подобные изменения будут зависеть от развития муниципальных моделей системы первой медицинской помощи, имеющие ясные делегирующие полномочия, достаточные ресурсы и институционные возможности для воплощения в жизнь.
Выводы: Развитие систем городского здравоохранения и стратегии вакцинации необходимы как на региональном, так и на государственном уровне как реакция на быстрые демографические изменения, социальное преобразование и растущий риск эпидемии.

Translated from English version into Russian by Elena Chavykina, proofread by Gunel Huseynbayova, through

Servicios de salud e inmunización para los pobres de zonas urbanas en países seleccionados de Asia

John Grundy, Xiaojun Wang, Kunihiko Chris Hirabayashi, Richard Duncan, Dexter Bersonda, Abu Obeida Eltayeb, Godwin Mindra, Robin Nandy

RESUMEN

Contexto: Asia es una región que se está urbanizando rápidamente. Si bien la salud urbana en general está por encima de los estándares de salud rural, también hay focos de profunda desventaja social y sanitaria dentro de los barrios marginales urbanos y las zonas periurbanas, que representan un mayor riesgo para la salud pública. Con un enfoque en las enfermedades que se pueden evitar mediante vacunación y en la cobertura de inmunización, este comentario describe y analiza las fortalezas y debilidades de la estrategia existente de inmunización y salud urbana, con el fin de recomendar direcciones estratégicas para mejorar el acceso a la inmunización y los servicios relacionados de salud materna e infantil en las zonas urbanas de todo el país. Los temas tratados en este comentario se basan en los hallazgos de los estudios de casos de países publicados por el Fondo Internacional de de las Naciones Unidas para la Infancia sobre el tema de la inmunización y los servicios relacionados de salud para los pobres urbanos en Camboya, Indonesia, Mongolia, Myanmar, Filipinas y Vietnam.
Desarrollo: Aunque la cobertura urbana general es mayor que la cobertura rural en los países seleccionados de Asia, también hay grandes disparidades en la cobertura entre grupos socioeconómicos dentro de las áreas urbanas. Acorde a estas brechas de cobertura, hay nueva evidencia de brotes de enfermedades que se pueden evitar mediante la vacunación en áreas urbanas. En respuesta a este riesgo elevado para la salud pública, se han producido algunas innovaciones prometedoras en la estrategia operativa en entornos urbanos, aunque la mayoría de estas iniciativas están relacionadas con proyectos y reciben financiación externa. Los temas críticos que necesitan atención en lo que refiere al acceso a los servicios de salud urbanos incluyen llegar a un consenso sobre la responsabilidad de la administración y los recursos de la estrategia, y la inclusión de un abordaje de la pobreza urbana dentro de los procedimientos de planificación y presupuesto de los ministerios de salud y los gobiernos locales. También se requiere el avance urgente de las asociaciones locales y de las estrategias de participación

comunitaria para informar los enfoques operativos para las poblaciones socialmente marginadas. Tales desarrollos dependerán del avance de modelos municipales de atención primaria de salud que tengan delegaciones claras de autoridad, recursos adecuados y capacidades institucionales para implementar.

Conclusiones: El desarrollo de los sistemas de salud urbanos y de la estrategia de inmunización es necesario a nivel regional y nacional para responder al rápido cambio demográfico, a la transición social y al aumento del riesgo epidemiológico.

Translated from English version into Spanish by Natalia Victoria Gómez, proofread by Maria Paula Gorgone, through

TRANSLATORS
WITHOUT BORDERS
